# Editorial: Methods and Protocols in Developmental and Reproductive Toxicology

**DOI:** 10.3389/ftox.2022.948103

**Published:** 2022-07-20

**Authors:** Karin Sørig Hougaard, Terje Svingen

**Affiliations:** ^1^ National Research Center for the Working Environment, Copenhagen, Denmark; ^2^ Department of Public Health, Faculty of Health and Medical Sciences, University of Copenhagen, Copenhagen, Denmark; ^3^ National Food Institute, Technical University of Denmark, Kgs. Lyngy, Denmark

**Keywords:** new approach methodologies (NAM), chemical test method, adverse outcome pathway (AOP), mechanisms, predictive toxicity assay, development

Undisturbed reproductive development is a prerequisite for life-long reproductive health. Many stages of perinatal life are vulnerable to disturbances such as exposure to environmental chemicals and drugs that can interfere with molecular and cellular processes. Of concern is the enormous number of chemicals and pharmaceuticals in existence and for which humans and wildlife can be exposed through their life cycles (Rockström et al., 2009; Svingen and Vinggaard, 2016; Hougaard, 2021).

Although there are international regulations and guidelines in place for testing and assessing chemical substances for their potential toxicity, they are in many ways inadequate in dealing with the vast number of chemicals in use coupled with the myriad of potential effect they can have on complex living organisms. This challenge is becoming even more pressing with modern toxicology moving towards higher dependence on alternative test methods rather than more traditional large-scale animal toxicity testing for screening and prioritizing chemical substances of concern (Pistollato et al., 2021). This is not least important in hypothesis-driven testing, or predictive toxicology, where knowledge about toxicological mechanisms and measurable key events increasingly feed into causal adverse outcome pathways (AOPs) (Ankley & Edwards, 2018; Audouze et al., 2021; Paini et al., 2022; Svingen et al., 2022). Once robust AOPs have been developed, chemicals can increasingly be assessed using alternative methods; in essence reducing the reliance on more traditional *in vivo* (animal) protocols. Thus, to ensure a safe environment for future generations, we need methods that can cut down on resources needed to carry out scientific research and enable hazard identification and safety assessment based on limited, or even no testing, in complex biological systems. The 21st Century has witnessed great strides towards this endeavor and this development will undoubtedly continue to move in the right direction. Yet, limitations with reductionist methodologies must simultaneously be considered when devising testing strategies relying on alternative test methods to properly account for emergent properties in complex organisms (Svingen, 2022).

Standardized and validated protocols are critically important in regulatory toxicology. However, formal validation and acceptance of new methods for hazard assessment, such as under the Organization for Economic Co-operation and Development (OECD), is laborious and time-consuming. Common guidelines for the reporting of experimental results are also very useful, but only if endorsed by the scientific community. Here, scientific journals play a role in providing a forum where scientists across sub-disciplines of toxicology can share methods and protocols that are tried and tested. This can encourage dialogue on ways by which toxicological research is conducted. On this background, we invited contributions on methods and protocols in developmental and reproductive toxicology to hopefully in the longer term improve our capacity to test and assess chemical substances in a reproducible and widely accepted manner.

This Research Topic collected four method papers addressing reproductive and developmental toxicity.

The first paper Hoyberghs et al. deals with optimization of the Zebrafish Embryo Developmental Toxicity Assay (ZEDTA), specifically on the optimal concentration of (DMSO), which is often used as a chemical solvent. Since high concentrations of DMSO is toxic to zebrafish larvae and embryos, it is imperative to keep DMSO concentrations below toxic levels when testing for potential effects of chemicals to avoid false positives. The maximum DMSO concentration of 0.01% that is recommended by the OECD may, however, in many instances be too low to dissolve test compounds. In contrast to OECD recommendations, this study shows that a DMSO concentration up to 1% is non-toxic in the ZEDTA assay Hoyberghs et al., a finding that will have direct impact on how the assay is performed and which chemicals that can be tested.

The second paper Garner et al. addresses how the isolated rat placenta can be used to assess fetoplacental hemodynamics. The *ex vivo* application of isolated placentas is well described for both animals and humans in the assessment of passage of chemicals between the maternal and fetal compartment. However, perturbations in for instance placental blood flow might lead to poor health outcomes for both the dam and the fetus. This paper describes a technique that allows for control of inflow pressure and analysis of resulting placental outflow pressure and flow in rat placentas by use of a modified pressure myography chamber. Hence, the model allows for characterization of changes in placental hemodynamics, for example as a consequence of chemical exposure during gestation Garner et al.

The third paper Onoda et al. reports on a novel method for detecting brain perivascular injuries, whereby periodic acid-schiff is combined with immunohistochemical staining in a double-staining protocol to increase sensitivity. Brain development is sensitive to perturbation by chemical exposures and the authors have previously shown brain perivascular tissues, and macrophages, to be particularly sensitive to maternal nanoparticle exposure during gestation (Onoda et al., 2017a; Onoda et al., 2017b). The authors propose that their improved staining method of tissues in close proximity to brain blood vessels can be used to evaluate developmental neurotoxicity Onoda et al.

The final paper Asimaki et al. describes a method for application in screening of chemicals for female reproductive toxicity, the bovine model of *in vitro* oocyte maturation with subsequent production of embryos, including events in oocyte maturation and acquisition of developmental competence. The model includes the *in vitro* maturation of cumulus-oocyte complexes followed by *in vitro* fertilization by co-incubation of oocytes with sperm, and subsequent *in vitro* embryo culture until the blastocyst stage. Endpoints represent important aspects of oocyte maturation and embryo development such as nuclear maturation, mitochondrial redistribution, cumulus cell expansion, apoptosis, and steroidogenesis, and in fertilized oocytes, embryo cleavage and blastocyst rates Asimaki et al.

The submitted papers on methods and protocols in developmental and reproductive toxicology describe both existing methods that were adapted for specific purposes, as well as new methods addressing novel aspects of developmental toxicity testing. Hopefully, they will in the longer term improve assessment of chemical substances within this field toxicology.
